# Recent progress of poly(glycerol adipate)-based network materials toward tissue engineering applications

**DOI:** 10.3389/fbioe.2024.1447340

**Published:** 2024-09-17

**Authors:** Anna Kłusak, Małgorzata Anna Gazińska

**Affiliations:** Department of Polymer Engineering and Technology, Faculty of Chemistry, Wrocław University of Science and Technology, Wrocław, Poland

**Keywords:** poly(glycerol adipate) elastomer, poly(glycerol adipate)-based network, chemical crosslinking, photo-crosslinking, cytotoxicity, degradation, linear viscoelastic properties, soft tissue regeneration

## Abstract

Poly(glycerol adipate) (PGA) is one of the aliphatic polyesters of glycerol. The most studied biomedical application of poly(glycerol adipate) is the use of its nanoparticles as drug delivery carriers. The PGA prepolymer can be crosslinked to network materials. The biomedical application of PGA-based network materials has largely remained unexplored till recently. The PGA-based network materials, such as poly(glycerol sebacate) elastomers, can be used in soft tissue regeneration due to their mechanical properties. The modulus of elasticity of PGA elastomers is within the range of MPa, which corresponds to the mechanical properties of human soft tissues. This short review aims at briefly summarizing the possible applications of PGA-based elastomers in tissue engineering, as indicated in recent years in research publications.

## Introduction

Tissue engineering is an interdisciplinary scientific field that has been widely developed in recent years. Its main goal is to rebuild and regenerate damaged tissues and internal organs or create entirely new tissues. Materials that are commonly used in tissue engineering need to be biocompatible, designed for long-term contact with living tissue and bodily fluids, without causing negative effects on the organism. They should also exhibit desired bioactivity and appropriate mechanical properties, all tailored to specific applications, and also be biodegradable. Various groups of materials, such as polymers, ceramics, or polymer–ceramic composites, are being developed for tissue engineering (Qu et al., 2019; Mazzoni et al., 2021; Farag, 2023; Liu et al., 2023; Szwed-Georgiou et al., 2023). Some of the most commonly used biodegradable polymers are aliphatic polyesters, i.e., polylactide, polyglycolide, and polycaprolactone. They are among thermoplastic polymers, are easily processible and have good mechanical resistance and high durability under various conditions (Asghari et al., 2017). Elastomeric polymers are most suitable for soft tissue engineering (Li et al., 2012; Coenen et al., 2018). Glycerol-based aliphatic polyesters are a group of synthetic elastomers (Shukla, 2022). These include poly(glycerol sebacate) (Piszko et al., 2021; Sha et al., 2021), poly(glycerol citrate) (Gadomska-Gajadhur et al., 2021; Wrzecionek et al., 2021), poly(glycerol itaconate) (Kolankowski et al., 2022), and poly(glycerol adipate) (PGA). In this group, the poly(glycerol sebacate) receives the most attention in tissue engineering (Kemppainen and Hollister, 2010; Rai et al., 2012; Zaky et al., 2014; 2017; Liang et al., 2020; Wrzecionek et al., 2024).The PGA-based elastomers are less extensively studied. Drug release and delivery is a widely described topic concerning the application of PGA as a biomaterial (Puri et al., 2008; Zamboulis et al., 2019; Vestri et al., 2020; Alaneed et al., 2021; Jacob et al., 2023). Swainson et al. (2019a) and Parshad et al. (2021) have compiled relevant data on PGA from the literature.

**FIGURE 1:**
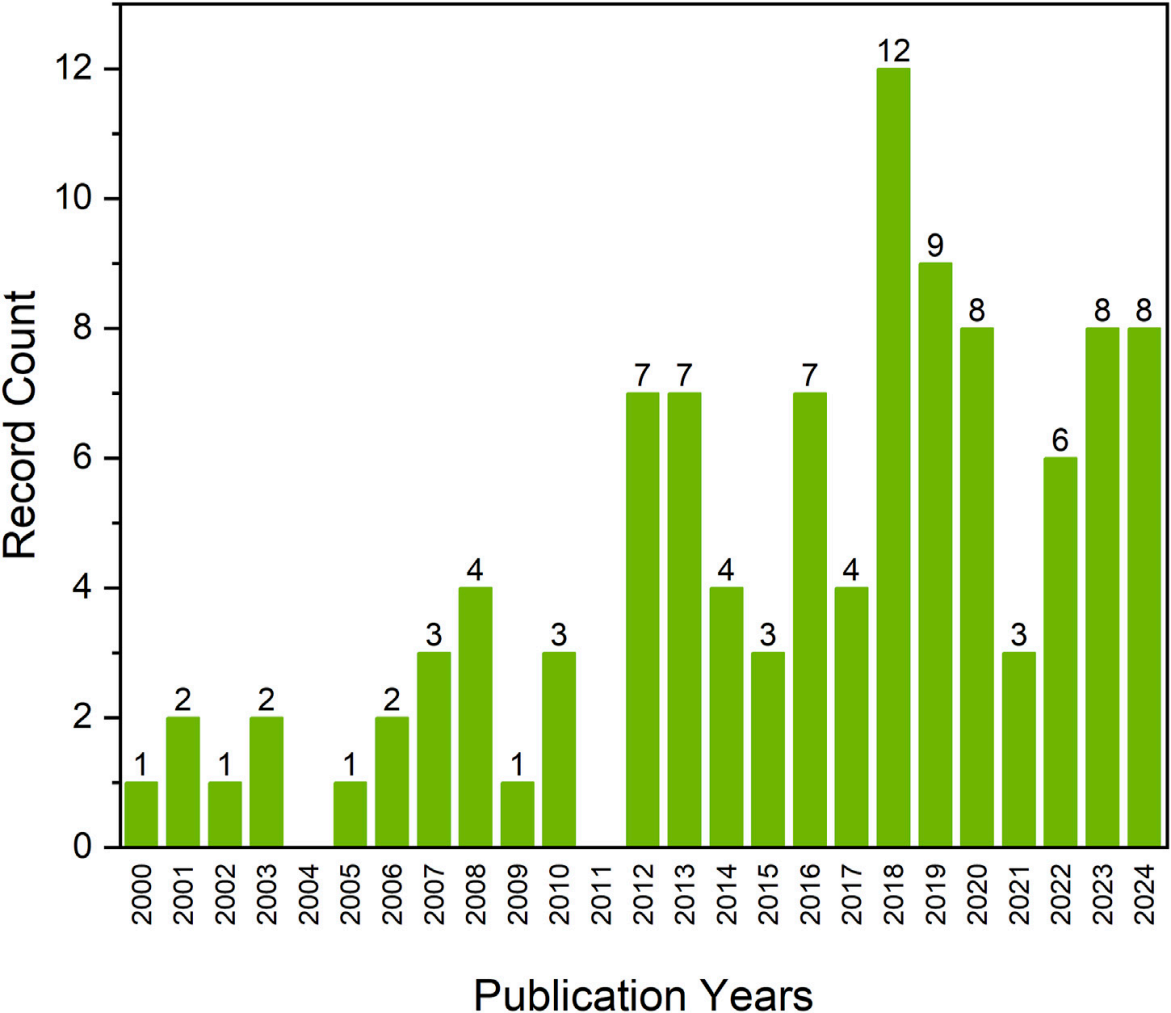
Number of scientific publications regarding poly(glycerol adipate) in years 2000–2024 based on Web of Science Core Collection (date of the analysis 31.05.2024).

Thanks to the elastomer formation by crosslinking of poly(glycerol adipate) prepolymer, first described by Nagata et al. (1996), Kiyotsukuri et al. (1994), and by Navarro et al. (2017), a new direction for medical applications of PGA—tissue engineering—could be developed. A yearly overview of the number of publications on poly(glycerol adipate) between 2000 and 2024 shows that the interest in the topic peaked in 2018 (Figure 1), which could be related to the work by Navarro et al. (2017) on network formation from poly(glycerol adipate) pre-polymer, beginning the development of a new direction of PGA elastomer in soft tissue engineering.

This short review intends to briefly summarize the current state of PGA-based network materials and envisage their development in tissue engineering. The characteristics of PGA-based elastomers in terms of crosslinking, cytotoxicity, degradation, mechanical properties, and results of the *in vitro* studies are collected. Because the properties of the PGA elastomer depend on those of the prepolymer, this review starts by focusing on the synthesis methods and properties of the PGA prepolymers.

### Synthesis and properties of the PGA prepolymer

Poly(glycerol adipate) is a biodegradable aliphatic polyester, which is not commercially available. The synthesis of PGA involves the reaction of glycerol with a suitable co-monomer, such as divinyl adipate (Taresco et al., 2016a; Alaneed et al., 2021; Olalla et al., 2022; Hevilla et al., 2023b; 2023a), dimethyl adipate (Naolou et al., 2013b; Bilal et al., 2019; Zamboulis et al., 2019), or adipic acid (Iglesias et al., 1999; Naolou et al., 2013b; Zhang et al., 2014; Navarro et al., 2017; Zeng et al., 2020). The process produces viscous transparent liquids called prepolymers, due to their relatively low molar masses of a few to several kDa (Kline et al., 1998; Kumar et al., 2003). Various synthesis conditions yield linear or hyperbranched PGA prepolymers (Zamboulis et al., 2019). The synthesis can be carried out in the absence of a catalyst, with metallic catalysts, or in the presence of specific enzymes. If the process is catalyzed by lipase extracted from the B fraction of *Candida antarctica*, immobilized on a macroporous acrylic resin (Novozym 435), it can be performed under milder conditions, and it can control regioselectivity, generating low toxicity (Kline et al., 1998; Taresco et al., 2016a).

The most popular and widely published approach to synthetizing the PGA prepolymer is the reaction involving equimolar amounts of divinyl adipate and glycerol carried out in tetrahydrofuran and catalyzed by lipase B (Kline et al., 1998; Naolou et al., 2013a; Taresco et al., 2016a; Bilal et al., 2017; 2019; Swainson et al., 2019a; Zamboulis et al., 2019; Jacob et al., 2021; Olalla et al., 2022; Hevilla et al., 2023a; Gazińska and Krokos, 2024; Gazińska et al., 2024). This approach is advantageous, and the by-product is vinyl alcohol that readily tautomerizes to acetaldehyde, which evaporates at the reaction temperature, rendering the reaction irreversible. Jacob et al. (2021) improved this approach by performing the synthesis in a solvent derived from renewable resources, 2-methyl tetrahydrofuran, in place of fossil-based THF.

PGA is an amorphous and hydrophilic polymer; however, it is insoluble in water despite the presence of hydroxyl groups (Taresco et al., 2016a; Zamboulis et al., 2019). A great advantage of PGA is the presence of the pendant hydroxyl group, allowing functionalization of the PGA prepolymer and producing modified polymeric materials with diverse properties (Weiss et al., 2018; Jacob et al., 2021; Steiner et al., 2021; Hevilla et al., 2023a; Suksiriworapong et al., 2023). The hydroxyl groups also enable crosslinking to achieve an elastomeric material.

### Crosslinking of the PGA prepolymer

In order to obtain an elastomeric material from the PGA prepolymer, it is necessary to crosslink it. The three main crosslinking methods are as follows: thermal-, chemical-, and photo-crosslinking. The first described method of PGA-based network formation was thermal crosslinking of the PGA prepolymer, presented by Kiyotsukuri et al. (1994). The PGA prepolymers were cast from the dimethylformamide solution and post-polymerized at various temperatures and times, which yielded networks in the form of transparent, flexible films insoluble in organic solvents. Nagata et al. (1996) described the PGA prepolymer obtained by melt condensation of glycerol and adipic acid and further followed by thermal crosslinking at 200°C for 90 min in a nitrogen atmosphere. Navarro et al. (2017) presented the formation of PGA elastomers by curing of the PGA prepolymer layer in an oven at 120°C for 3 and 5 days. Gazińska et al. (2024) carried out thermal crosslinking of a PGA prepolymer in a circulating air dryer at 110°C for 5 days.

Chemical crosslinking, another method of elastomer formation from the PGA prepolymer, requires the use of a chemical crosslinking agent reacting with the free hydroxyl groups of glycerol or vinyl or carboxyl end groups, and it results in the formation of covalent bonds between the chains. The crosslinking agent can be selected to introduce a beneficial effect on the target application of the elastomeric material, in addition to effective crosslinking. Such an approach was used in our research on PGA-based elastomers (Gazińska and Krokos, 2024). We used a diisocyanate derivative of L-lysine (LDI) as the crosslinking agent. L-lysine is known from promoting osteoblast adhesion and proliferation (Liuyun et al., 2016; Yoshikawa et al., 2015), and L-lysine increases the osteogenic potential of bone stem cells (Liuyun et al., 2016). As a first step, the prepolymer was modified with LDI and then cured at 35°C for 3 and 7 days depending on the amount of LDI. The higher crosslinking agent content resulted in a higher degree of crosslinking and increased the rubbery plateau modulus. The main advantages of the crosslinking method of the PGA prepolymer with LDI were as follows: (i) reduction in the curing stage temperature to 35°C (rather than above 100°C that occurs in thermal crosslinking) and (ii) the introduction of the L-lysine moiety. Benefits of using L-lysine derivatives for network formation require confirmation in biological studies.

Formation of the PGA-based network through a chemical reaction pathway was reported by Alaneed et al. (2021). The PGA-based network in the form of gel was formed by the Michael addition reaction between vinyl end groups of the PGA prepolymer and primary amine groups introduced in the acylation reaction of PGA prepolymer with 6-(Fmoc-amino) hexanoic acid, followed by the removal of Fmoc protecting groups. The advantages of the presented approach are the mild and catalyst-free conditions. The authors indicated potential pharmaceutical applications of the formed hydrogel as a carrier for sustained release of hydrophilic drugs and biodegradable implants for controlled drug delivery.

Hevilla et al. (2023a) presented a photo-crosslinking method of PGA network formation from PGA functionalized with vinyl methacrylate (VMA). In order to crosslink PGA, VMA and the photoinitiator IRGACURE 369 were added. Crosslinking was carried out in the presence of 2-(4-methylthiazol-5-yl) ethyl methacrylate under a UV lamp for 10 min. Hevilla et al. (2023b) showed that a longer crosslinking time did not increase the crosslinking density. They considered a time of 10 min to be optimal. The results of both studies clearly indicate that the functionalization of PGA with methacrylate groups allows it to be photo-crosslinked. The authors also pointed out that the resulting polymer networks can be manufactured in 3D printing technology to produce scaffolds such as prostheses, screws, or pins.

Thermal crosslinking of the PGA prepolymer requires a high temperature to initiate a polycondensation reaction that leads to a three-dimensional structure of the polymer material. Chemical- and photo-crosslinking methods enable the formation of networks at low temperatures, which makes it possible to introduce bioactive components into the network structure without their thermal degradation at the annealing stage.

### Cytotoxicity of PGA networks

Cytotoxicity is the ability of a specific agent to disrupt cell homeostasis, which can ultimately lead to dysfunction or death (Severin et al., 2017). This characteristic is an important aspect concerning various biomaterials intended for use in tissue engineering. So far, several *in vitro* studies have been published on cytocompatibility tests for PGA-based nanoparticles and nanoaggregates (Weiss et al., 2012; Zaky et al., 2014; Jacob et al., 2021; 2023) and for PGA-based network materials (Navarro et al., 2017; Hevilla et al., 2023a). Studies on PGA and nanoaggregates have shown that the nanometric particles are not cytotoxic. From the perspective of the application of PGA-based networks in tissue engineering, the results of the cytotoxicity of crosslinked PGA are crucial. Navarro et al. (2017) also conducted *in vitro* cytotoxicity studies on NIH 3T3 mouse embryonic fibroblasts. For this purpose, the PGA was modified with ethylene glycol, and then elastomeric films were produced from it, onto which mouse fibroblasts were seeded. The results showed that PGA elastomeric films were not cytotoxic to the cells, as the cells’ viability exceeded 70%. Navarro et al. (2017) examined the cell proliferation and indicated that mouse fibroblast cells as pluripotent cells seeded on the elastomer differentiated into neuroderivative dendritic cells. Hevilla et al. (2023a) investigated the behavior of human red blood cells (RBCs) in contact with photocured networks of methacrylated PGA. Toxicity tests were performed for 1 and 24 h by adding methacrylated PGA networks to RBC suspensions. The results indicated that the networks were not hemolytic, confirming they were not toxic to RBCs.

Most available publications focus on *in vitro* tests of PGA-based materials. In recent years, there have been studies that describe *in vivo* tests carried out on whole organisms. *In vivo* studies have the advantage of allowing us to observe how a given factor affects the entire organism, not only individual cells. *In vivo* studies were carried out only for PGA-based nanoparticles (Weiss et al., 2018; Jacob et al., 2023). Currently, there are no published *in vivo* test results for PGA-based network materials.

### Degradation of PGA networks

To properly and effectively apply PGA as a biomaterial, it is important to understand its degradation mechanism and identify its degradation products. For PGA-based network materials, knowledge about its degradation under the influence of various factors present in the human body is crucial. Understanding the degradation of PGA will also facilitate the design of materials based on it. In tissue engineering, the material lifespan is important. It must be longer than that of the material for drug release because it is intended to serve as a scaffold supporting tissue regeneration or even replacement. Navarro et al. (2017) conducted an *in vitro* degradation study of PGA elastomeric films incubated in a phosphate-buffered saline (PBS) solution at pH 7.4 at 37°C. After 60 days of incubation, there was a 31% mass loss for the PGA obtained from an equimolar ratio of adipic acid to glycerol and a 15% loss for the PGA obtained with excess adipic acid. The lower mass loss in the latter elastomer resulted from lesser free hydroxyl groups and a higher degree of crosslinking (Navarro et al., 2017). The results indicate the rate of PGA elastomer degradation, but they do not indicate degradation products.

Literature data focus on the enzymatic degradation of PGA nanoparticles because they have been mainly used in drug release (Taresco et al., 2016b; Swainson et al., 2019b). Swainson et al. (2019b) examined the degradation of PGA nanoparticles in detail under the influence of six enzymes present in the human body. The great advantage of this study is that the authors successfully identified PGA degradation products such as PGA dimers, adipic acid, and glycerol, which confirmed the formation of non-toxic compounds.

There are currently no data concerning the degradation products of PGA network materials. Especially in the case of chemically crosslinked and photo-crosslinked PGA network materials, the degradation products still need to be identified and verified in terms of their toxicity and effects of cumulation *in vivo*.

### Mechanical properties of PGA networks

The mechanical properties of the PGA networks depend on the prepolymer synthesis conditions, prepolymer microstructure, and crosslinking method (Table 1).

**TABLE 1:** Summary of literature data on the mechanical properties of PGA-based network materials.

PGA – based crosslinked materials	Crosslinking method	Mechanical properties	References
PGA elastomers from PGA prepolymers synthesized at glycerol:adipic acid ratios of 1:1 1:0.6 PGA elastomers modified with ethylene glycol (glycerol:adipic acid:ethylene glycol) 1:0.75:0.25 1:0.5:0.50 1:0.25:0.75	Thermal crosslinking at 120°C for 3 and 5 days	Young Modulus 2.41 ± 0.09 MPa 5.00 ± 0.72 MPa 1.18 ± 0.18 MPa 0.43 ± 0.01 MPa 0.07 ± 0.02 MPa	Navarro et al. (2017)
PGA-based networks obtained from mixtures of the macromer PGA functionalized with vinyl methacrylate (PGA-MA) and photocurable methacrylic monomer (MTA) PGA-MA PGA-MA+5 wt% MTA PGA-MA+10 wt% MTA PGA-MA+15 wt% MTA	Photocrosslinking with the UV lamp for 10 min in the presence of photoinitiator (Irgacure®369) Photocrosslinking of mixtures of photocurable methacrylic monomer and a photocurable telechelic macromer based on poly (glycerol adipoate) functionalized with vinyl methacrylate	Microhardness 1.17 ± 0.10 MPa 1.82 ± 0.42 MPa 2.38 ± 0.44 MPa 2.71 ± 0.30 MPa	Hevilla et al. (2023a)
PGA elastomers from PGA prepolymers synthesized for 8 and 24 h PGA8 PGA24	Thermal crosslinking of PGA prepolymer at 110°C for 5 days	Rubbery plateau modulus at 37°C 116 kPa 503 kPa	Gazińska et al. (2024)
Poly (glycerol adipate urethane) elastomers PGAU-LDI50% PGAU-LDI100%	Chemical crosslinking of PGA prepolymer with L-lysine diisocyanate	Rubbery plateau modulus at 37°C 278 kPa 1.55 MPa	Gazińska and Krokos (2024)

The tensile properties of PGA elastomers and elastomers of PGA modified with varying ethylene glycol contents were evaluated by Navarro et al. (2017). PGA elastomers were obtained by thermal crosslinking at 120°C of a PGA prepolymer. Young’s modulus of the elastomers ranged from 0.07 to 8.33 MPa, depending on the curing conditions, monomer ratio, and addition of ethylene glycol. The authors conclude that Young’s modulus is within the range of various soft tissues, such as nerve tissue, cartilage, and skin. They conclude that, based on the evaluation of *in vitro* results, Young’s modulus of elastomers is one of the factors that regulates the differentiation of NIH/3T3 cells. The authors go on to hypothesize that Young’s modulus of elastomers plays a central role in cell differentiation.

Hevilla et al. (2023a) investigated the microhardness of PGA-based networks in the form of films obtained by photo-crosslinking of mixtures of the photocurable methacrylic monomer and a photocurable telechelic macromer based on poly(glycerol adipate) functionalized with vinyl methacrylate. The microhardness increased form 1.17 ± 0.10 MPa to 2.71 ± 0.30 MPa as the amount of photocurable methacrylic monomer increased, which is due to an increase in the crosslinking density.

The natural response of living tissues to stress is not only purely elastic, but it also has a viscous component. We applied dynamic mechanical analysis (DMA) to evaluate the linear viscoelastic properties (LVE) that the PGA elastomers formed by thermal crosslinking of PGA prepolymers (Gazińska et al., 2024). The master curves of the storage and loss moduli were constructed at a reference temperature of 37°C. We highlighted that the frequency range of the rubbery state at 37°C is one of the key parameters determining the suitability of the elastomer in soft tissue engineering as the implant material should be in the rubbery state at working conditions. The rubbery plateau modulus of the PGA elastomers (116 kPa and 503 kPa) is within the range of the stiffness of soft tissues. We hypothesized that due to their similarity to osteoid, PGA-based elastomers could also be used for bone tissue regeneration.

DMA was also employed to study PGA-based elastomers in the form of poly(glycerol adipate urethane) (PGAU) networks obtained by chemical crosslinking of PGA prepolymer with a diisocyanate crosslinking agent (Gazińska and Krokos, 2024). The LVE properties of the composites of PGAU with unmodified and L-lysine-modified hydroxyapatite particles were evaluated. Depending on the amount of LDI, HAP, and surface modification, the rubbery plateau modulus ranges from 278 kPa to 3.98 MPa. The LDI content turned out as the most important among the tested factors. The addition of HAP particles modified with L-lysine favorably resulted in a shift of the glass transition toward higher frequencies and expansion of the frequency range of the rubbery state. The rubbery plateau modulus of the PGAU-based composites was within the stiffness range of soft tissues.

The described analysis of the mechanical properties of PGA-based network materials indicates that the mechanical properties could be regulated and tailored to specific soft tissues. It should be noted that the newer approach in tissue engineering does not focus on mechanical matching of the material to tissues but focuses on mechanical stimulation. Studies on the efficiency of the mechanotransduction effect on PGA-based network materials will be an interesting direction for future research.

## Discussion

Elastomeric polyesters such as PGA elastomers exhibit unique viscoelastic properties at body temperature conditions, which makes them applicable as effective scaffolds for soft tissue regeneration. PGA elastomer manufacturing involves the preparation of pre-polymers, followed by a crosslinking step. Among the available crosslinking methods, i.e., thermal-, chemical-, and photo-crosslinking, the most advantageous are chemical- and photo-crosslinking methods. By these methods, low aging temperature allows for the introduction of bioactive components into the volume of the material at the curing stage without the risk of their thermal degradation. The crosslinking step is essential as it enhances the material’s properties, ensuring that it adequately meets the requirements of the intended application. Crosslinking and network structure formation enable control over the mechanical properties, which is why PGA elastomers could be employed for the regeneration of soft tissues of varying stiffness. The challenge in tissue engineering is to produce a biocompatible biomaterial. In this regard, it is necessary to conduct *in vitro* and *in vivo* studies and determine the degradation products of chemically and photochemically crosslinked PGA materials and determine their interactions with the living tissues and the whole organism. Despite this challenge, if these criteria are met, such elastomers can prove valuable in an extensive range of clinically relevant applications. The publications cited in this review point to new directions of application of poly(glycerol adipate) as a material for tissue engineering.
